# Preservative Fluid

**Published:** 1883-10

**Authors:** 


### PRESERVATIVE FLUID.

The formula of a fluid for the preservation of animal tissue, which is said to equal the well-known Wickersheimer solution, is published by Dr. Wirodzeff of St. Petersburg. It is composed of thymol 5 parts, alcohol 45 parts, glycerine 2160 parts, water 1080 parts.—*Zahntechnische Reform.*

